# Supplementary material for: Morphology and Distribution of Antennal Sensilla in Three Species of Thripidae (Thysanoptera) Infesting Alfalfa Medicago sativa
Source: Insects. 2021 Jan 18;12(1):81. doi: 10.3390/insects12010081 (PMC7831480; doi:10.3390/insects12010081)
Supplement: Supplementary file 1 [file insects-12-00081-s001.pdf]

Supplementary Materials：

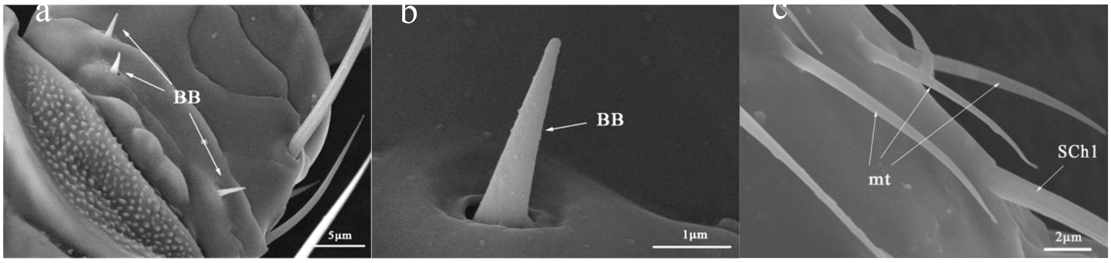

Figure S1. (a-b) Böhm bristle (BB) at the scape of *O. loti.* (c) Microtrichia (mt) at the flagellum Ⅱ of *O. loti.*

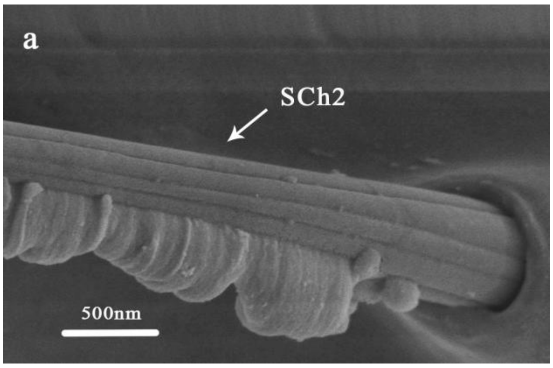

Figure S2. It was found that substances could be secreted in the longitudinal opening of SChⅡ in *Sericothrips kaszabi* by SEM.

Table S1. Mean(μm)±SE values of the length and width of the antennal segments of three species of thrips.

Antennal segment		*Odontothrips loti*		*Megalurothrips distalis*(N=9)	*Sericothrips kaszabi*(N=6)
		Females(N=10)	Males(N=10)		
Scape	Length	24.28±0.84	19.89±0.43	28.27±1.66	19.43±0.76
	Width	30.49±0.82	28.86±0.41	34.59±1.29	28.77±0.96
Pedicel	Length	40.27±1.02	35.96±0.21	40.38±1.53	33.11±1.53
	Width	27.14±0.89	24.22±0.22	30.94±0.83	27.57±0.67
Flagellum Ⅰ	Length	53.49±1.34	48.73±0.70	61.85±1.46	55.03±2.73
	Width	20.23±0.23	17.71±0.16	29.13±0.55	19.35±0.47
Flagellum Ⅱ	Length	56.51±0.56	51.07±0.86	73.05±1.63	53.95±2.16
	Width	19.77±0.26	18.11±0.27	27.40±0.36	17.67±0.22
Flagellum Ⅲ	Length	40.48±1.29	34.95±0.80	47.39±2.02	46.97±1.79

	Width	17.84±0.14	16.57±0.12	18.20±0.27	16.81±0.32
Flagellum Ⅳ	Length	60.01±1.00	52.15±0.76	65.29±2.14	53.58±1.71
	Width	18.82±0.31	17.39±0.31	20.90±0.26	16.02±0.47
Flagellum Ⅴ	Length	11.66±0.47	9.92±0.25	17.84±0.60	11.58±0.70
	Width	7.61±0.31	8.42±0.23	9.95±0.26	6.60±0.16
Flagellum Ⅵ	Length	17.04±0.40	14.32±0.25	23.07±0.79	15.79±0.44
	Width	5.61±0.07	5.95±0.19	6.61±0.16	5.00±0.13
Total of antennae	Length	298.13±3.27	261.27±2.07	355.12±6.96	290.61±9.33
